# Paternal Deprivation Alters Neural Stem Cells Proliferation and Epigenetic Histone Modifications in the Neurogenic Niches of Adult Prairie Voles

**DOI:** 10.3390/ijms27031556

**Published:** 2026-02-05

**Authors:** Dulce María Arzate, Guadalupe Denisse Rivera-Bautista, Giovanna Fregoso-Barrera, Analía E. Castro, Francisco Camacho, Daniela Ávila-González, Raúl G. Paredes, Néstor F. Díaz, Wendy Portillo

**Affiliations:** 1 Departamento de Neurobiología Conductual y Cognitiva, Instituto de Neurobiología, Universidad Nacional Autónoma de Mexico (UNAM), Campus Juriquilla, Queretaro 76230, Mexico; 2 Escuela Nacional de Estudios Superiores, Universidad Nacional Autónoma de Mexico (UNAM) Unidad Juriquilla, Queretaro 76230, Mexico; 3 Departamento de Fisiologia y Desarrollo Celular, Instituto Nacional de Perinatología, Ciudad de Mexico 11000, Mexico

**Keywords:** adult neurogenesis, neurogenic niches, epigenetic marks, monoparental family, pair-bonding

## Abstract

Paternal deprivation has behavioral, neurochemical, and neuroendocrine consequences in adulthood. Socially monogamous prairie voles (*Microtus ochrogaster*) raised only by the mother (monoparental care, MP) showed low levels of alloparental behavior and delayed pair bonding formation in adulthood compared to those raised by both parents (biparental care, BP). However, the effects of paternal deprivation on adult neurogenesis and the epigenetic mechanisms involved remain to be elucidated. Here, we focused on the impact of MP rearing on neural stem cells (NSCs) proliferation under basal conditions and in response to cohabitation with the sexual partner during pair bonding formation. At basal conditions, we found a significant decrease in the number of new proliferative NSCs (BrdU^+^/SOX2^+^) in male and female MP voles compared to BP animals in the subventricular (SVZ) and subgranular zone (SGZ). After 24 h of cohabitation, in MP males, there was an increase in the number of newborn cells in the SVZ but not in the SGZ. However, this increased proliferation was lower than in BP males. In females, we did not observe significant differences compared to controls. Finally, we evaluated the enrichment of H3K4me3 (activation) and H3K27me3 (silencing) epigenetic marks in the new cells, finding differences between rearing systems and sexes.

## 1. Introduction

Long-term pair relationships constitute a central dimension of human social behavior and exert profound influences on physical and psychological health [1]. Monogamy, a reproductive strategy defined by cooperation and biparental care, is additionally associated with reduced infanticide [2]. Despite extensive behavioral descriptions, the cellular and molecular mechanisms underlying the formation and maintenance of a socio-sexual bond remain only partially understood.

The prairie vole (*Microtus ochrogaster*) is a socially monogamous rodent species widely used to study pair bonding. Adult females and males form a stable pair bond after 24 h of cohabitation without physical contact, or after approximately 6 h of sexual interaction [3]. Early-life social environment critically shapes these behaviors: voles reared in monoparental (MP) or single-parent families exhibit delayed pair-bonding and provide reduced parental care [4,5]; accompanied by lower dopamine utilization in the nucleus accumbens, a key node in the reward circuit [6]. Nevertheless, MP reared voles retain innate opposite-sex olfactory preferences and show no deficits in odor detection, olfactory discrimination, or sexual behavior [6].

Neuroimaging studies after 24 h of cohabitation with the sexual partner have identified a “huddling network” involved in pair-bond formation, including the olfactory bulb (OB), dentate gyrus (DG), lateral septum, nucleus accumbens, medial prefrontal cortex, medial amygdala, and ventral pallidum [7]. The OB establishes connections with central areas such as the amygdala, the medial preoptic area, and the ventromedial hypothalamus, which are involved in the display of sexual behavior, pair bonding formation, aggression, parental care, and social behaviors [8,9]. Sexual behavior depends on the integrity of the olfactory system, since both males [10] and females [11] with bilateral bulbectomy or vomeronasal organ removal [12,13] do not copulate or establish a partner preference. The hippocampus, a region involved in memory and learning, receives afferent projections from the OB via the lateral olfactory tract to the entorhinal cortex [14,15,16]. In turn, the entorhinal cortex sends input to the DG, which then relays information to the CA3 region, forming a circuit involved in pattern separation processes during memory formation [17].

Interestingly, adult rodents exhibit constitutive neurogenesis in some of these brain regions. Neural stem cells (NSCs) in the subventricular zone (SVZ) generate newborn neurons that migrate to the OB, whereas NSCs in the subgranular zone (SGZ) of the hippocampal DG produce granule neurons throughout adulthood. A third neurogenic niche has been described in the median eminence of the hypothalamus, where tanycytes contribute new orexigenic and anorexigenic neurons [18,19,20,21,22,23,24]. Growing evidence suggests a role for adult neurogenesis in socio-sexual interactions.

In prairie voles, Liu and colleagues showed that exposure to opposite-sex odor increases the number of newborn cells in the main and accessory OB in both sexes, without altering DG proliferation; additionally, in females, exposure to male bedding increases cell proliferation in the amygdala. These effects depend on intact olfactory bulbs, as bulb lesions reduced the number of new cells in both the amygdala and DG [25]. Moreover, in females, multisensory socio-sexual cues (including olfactory cues) can induce sexual receptivity, increase SVZ proliferation, and bias neuronal commitment [26]. Consistent with these findings, our group reported that social exposure to a female without physical contact increases the number of newborn cells in the dorsal region of the SVZ and DG of male prairie voles [27]. Furthermore, social exposure to an opposite-sex conspecific or engagement in sexual activity increases NSCs proliferation in the SVZ and SGZ yielding a greater number of neurospheres *in vitro* [28]. NSCs derived from animals with socio-sexual experience also generate more neuroblasts in vivo [27] and *in vitro* [28]. Independent studies have shown that cohabitation with mating, leading to pair-bond formation, increases the number of newborn neurons in the OB and hippocampus [29]. In female voles, socio-sexual stimulation additionally augments proliferation in the amygdala and hypothalamus [30]. Collectively, these findings support the notion that adult neurogenesis contributes to the establishment of long-lasting pair bonds and is modulated by social exposure. Early rearing conditions also shape adult hippocampal neurogenesis in other monogamous vole species. In *Microtus mandarinus*, paternal absence during early postnatal life reduces adult hippocampal neurogenesis and impairs performance in a short-term social recognition task [31].

Adult neurogenesis is regulated by diverse intrinsic and extrinsic factors (reviewed in [32,33]). Epigenetic mechanisms, in particular, orchestrate NSC quiescence, activation and differentiation [34]. Active promoters and enhancers are marked by methylation of histone H3 at lysine 4 (H3K4me) [35], which are enriched at genes mediating neural progenitor differentiation and maturation [36,37]. In contrast, trimethylation of histone H3 at lysine 27 (H3K27me3) is associated with transcriptional repression [38]. The present study aimed to evaluate how MP rearing influences proliferative activity within the SVZ and SGZ of adult male and female prairie voles, both under basal conditions and during early cohabitation with a sexual partner. A second goal was to determine whether differences in newborn cell proliferation are accompanied by alterations in the enrichment of histone marks H3K4me3 and H3K27me3.

## 2. Results

### 2.1. Sexual Behavior of Biparental and Monoparental Reared Prairie Voles

To evaluate sexual behavior in biparentally (BP) and monoparentally (MP) reared prairie voles during cohabitation, we recorded latency and number of mounts, intromissions, and ejaculations. No statistically significant differences were found in any sexual behavior parameter between BP and MP males (Table 1). These findings are consistent with those previously reported by Valera-Marin and colleagues [6], indicating that MP rearing does not affect the expression of male sexual behavior.

Female prairie voles do not exhibit an estrous cycle comparable to that of rats and mice. Sexual receptivity is typically induced within the first 24–48 h of exposure to male odors [39,40,41]. In our study, experimental females were sexually naive, gonadally intact and did not receive hormonal treatment. As a consequence, only a subset of females allowed the male to mount (BP: 2 of 8 females; MP: 4 of 8 females), and no intromissions or ejaculations occurred.

### 2.2. Partner Preference Test Following 24 h of Cohabitation

Pair-bonding formation was assessed using the partner preference test [6]. We recorded time spent with the sexual partner and with a novel opposite-sex vole (stranger), as well as the number of huddling events. A preference index was calculated by dividing the time spent with the sexual partner by the total duration of the test (Table 2).

First, we compared the time spent with the sexual partner versus the stranger. MP females (t_(14)_ = 3.24, *p* = 0.005) and BP females (t_(14)_ = 8.54, *p* < 0.001) spent significantly more time with their sexual partner. In contrast, MP males (t_(18)_ = −2.22, *p* = 0.03) spent more time with the stranger, and BP males (t_(14)_ = −1.61, *p* = 0.12) did not show a significant preference.

When comparing time spent with the sexual partner between BP and MP males and females, we found a significant effect of sex (F_(1,30)_ = 20.92, *p* < 0.001), but no effect of rearing conditions (BP vs. MP: F_(1,30)_ = 0.181, *p* = 0.674) or sex x rearing interaction (F_(1,30)_ = 0.221, *p* = 0.642). Post hoc analysis revealed that BP females spent more time with their partner than BP males (*p* = 0.002) and MP females spent more time with their partner than MP males (*p* = 0.006).

Similarly, for time spent with the stranger, we observed effects of sex (F_(1,30)_ = 22.09, *p* < 0.001) but not rearing (F_(1,30)_ = 0.338, *p* = 0.565) or interaction (F_(1,30)_ = 0.398, *p* = 0.533). Females spent less time with the stranger vole than males (BP females vs. BP males, *p* < 0.001 and MP females vs. MP males, *p* = 0.006).

Significant differences were also found for the preference index [sex (F_(1,30)_ = 22.05, *p* < 0.001), with no effect of rearing (F_(1,30)_ = 0.318, *p* = 0.577) or interaction (F_(1,30)_ = 0.373, *p* = 0.546)]. Post hoc test reveals that females have a higher preference index than males (BP females vs. BP males, *p* < 0.001 and MP females vs. MP males, *p* = 0.006). Together, these results suggest that, although 24 h of cohabitation are generally sufficient for pair-bond formation [3,8,42], this process appears to occur more rapidly in females than in males.

Huddling, a key behavioral indicator of pair bonding, was analyzed using a chi-square test of independence to examine the relationship between rearing and engagement in huddling with the sexual partner. In females, this relation was significant (X^2^_(1,16)_ = 6.3492, *p* = 0.0117), with more BP females engaging in huddling than MP females. In males, although a higher proportion of BP males displayed huddling with their sexual partner, the difference compared to MP males was not significant (X^2^_(1,16)_ = 0.18, *p* = 0.6713).

### 2.3. Proliferative Activity in Adult Neurogenic Niches

Newborn cells (BrdU^+^) and proliferating NSCs (BrdU^+^/SOX2^+^) were quantified in the SVZ and SGZ under basal conditions (Control groups) and after 24 h of cohabitation (Cohabitation groups).

#### 2.3.1. Cell Proliferation Levels in the SVZ of Male Prairie Voles

**Figure 1:**
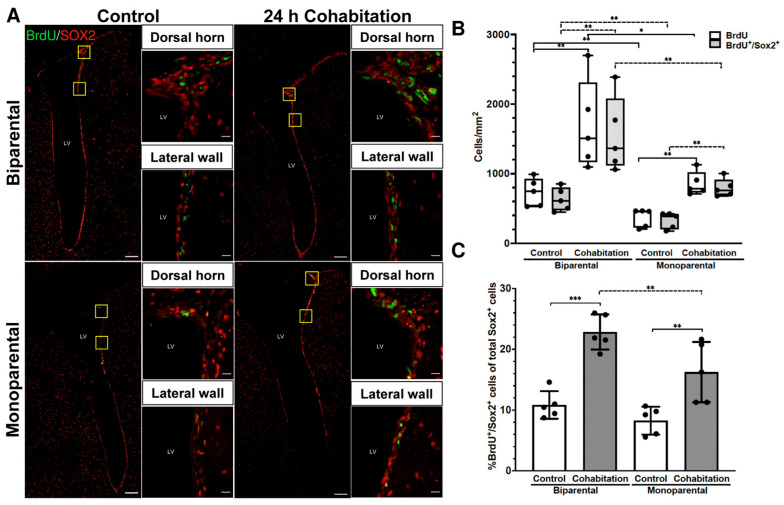
Cell proliferation levels in the SVZ of male prairie voles. (**A**) Representative photomicrographs of BrdU^+^/SOX2^+^ cells in the SVZ of BP and MP reared prairie voles. Scale bar panoramic image 100 μm; inset 10 μm. (**B**) Quantification of BrdU^+^ and BrdU^+^/SOX2^+^ cells per mm^2^. (**C**) Percentage of BrdU^+^/SOX2^+^ cells (proliferative NSCs) relative to total SOX2^+^ cells (NSCs). Significance level * *p* < 0.05, ** *p* < 0.01, *** *p* < 0.001.

Representative photomicrographs of BrdU^+^/SOX2^+^ cells in BP and MP voles from Control and Cohabitation groups are shown in Figure 1A.

We found a significant decrease in basal cell proliferation in the MP Control group compared with the BP Control group (*p* = 0.008; Figure 1B). This indicates that basal cell proliferation levels differ between BP and MP males, which may ultimately lead to reduced neurogenesis and influence the response of MP voles to socio-sexual stimuli.

Comparison between BP and MP males in the Cohabitation groups demonstrates an increase in the number of BrdU^+^ cells relative to their respective Controls (H_(3)_ = 15.937, n = 5, *p* = 0.001). Significant differences were detected between BP Control group and BP Cohabitation groups (*p* = 0.008) and between MP Control and MP Cohabitation groups (*p* = 0.008; Figure 1B). Although MP males exhibited an increase in cell proliferation after cohabitation with a female, this increase was significantly lower than that observed in BP males (BP Cohabitation vs. MP Cohabitation, *p* = 0.016). These results indicate that socio-sexual stimulation promotes SVZ cell proliferation in both BP and MP males, but the response is stronger in BP voles.

To identify the NSC population, we performed SOX2 immunodetection. No significant differences were found in the number of SOX2^+^ cells per mm^2^ between groups [rearing: (F_(1,16)_ = 4.426, *p* = 0.052); treatment factor (F_(1,16)_ = 1.313, *p* = 0.269)], suggesting that the total NSC population in the SVZ is similar in MP and BP male voles.

We then quantified proliferative NSCs (BrdU^+^/SOX2^+^ cells per mm^2^; H_(3)_ = 16.554, n = 5, *p* < 0.001; Figure 1A,B). BP Cohabitation males showed higher number of BrdU^+^/SOX2^+^ cells than BP Controls (*p* = 0.007, left gray boxes in Figure 1B), and MP Cohabitation males showed higher number than MP Controls (*p* = 0.006, right gray boxes in Figure 1B). Under basal conditions, BP Control males had more BrdU^+^/SOX2^+^ cells than MP Control males (*p* = 0.008, Figure 1B) and BP Cohabitation males had more than MP Cohabitation males (*p* = 0.008, Figure 1B).

Consistent with these observations, we found significant effects of both rearing (F_(1,16)_ = 9.696, *p* = 0.007) and treatment (F_(1,16)_ = 45.98, *p* < 0.001) on the percentage of SOX2^+^ cells that incorporated BrdU (proliferating NSCs; Figure 1C). Fisher LSD test showed an increase in BP Cohabitation vs. BP Control group, MP Cohabitation vs. MP Control and BP Cohabitation vs. MP Cohabitation groups. No significant differences were observed between BP Control and MP Control groups in the proportions of BrdU^+^/SOX2^+^ cells (Figure 1C).

#### 2.3.2. Cell Proliferation Levels in the SGZ of Male Prairie Voles

**Figure 2:**
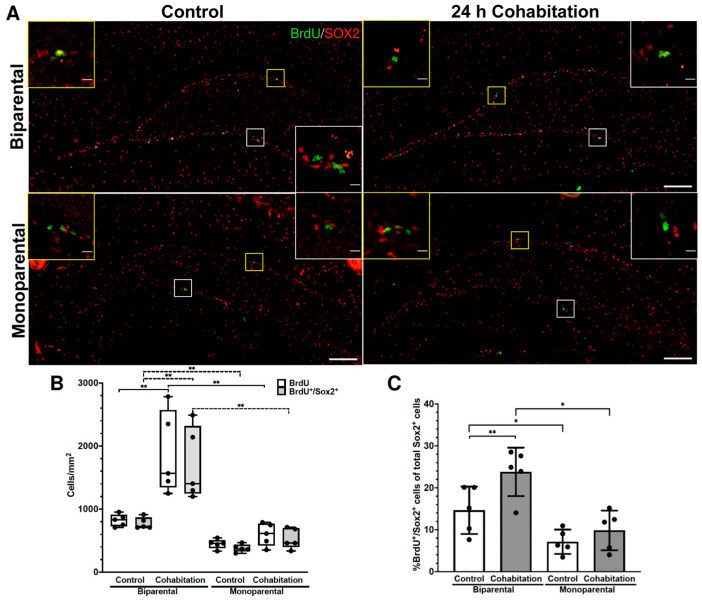
Cell proliferation levels in the SGZ of male prairie voles. (**A**) Representative photomicrographs of BrdU^+^/SOX2^+^ cells in the SGZ of BP (top) and MP (bottom) males. Yellow insets show cells in the suprapyramidal blade; white insets show the infrapyramidal blade. Scale bar in panoramic image 100 μm, inset 10 μm. (**B**) Quantification of BrdU^+^ and BrdU^+^/SOX2^+^ cells per mm^2^. (**C**) Percentage of BrdU^+^/SOX2^+^ cells (proliferative NSCs) from total SOX2^+^ cells. Significance level * *p* < 0.05 and ** *p* < 0.01.

In the SGZ of males, statistically significant differences were found in the number of BrdU^+^ cells per mm^2^ (H_(3)_ = 15.789, *p* = 0.001; Figure 2A,B). Post hoc analyses revealed that, under basal conditions, no significant differences were detected between BP and MP Control males. After cohabitation with a sexual partner, BP Cohabitation males showed increased BrdU^+^ cells compared with BP Controls, whereas no significant change was observed between MP Cohabitation and MP Control males (Figure 2B). Comparing BP Cohabitation vs. MP Cohabitation group revealed a significant difference, indicating a differential response to a socio-sexual stimulation in BP vs. MP males.

Similar results were obtained when evaluating the number of BrdU^+^/SOX2^+^ cells per mm^2^ (H_(3)_ = 15.891, *p* = 0.001; Figure 2A,B). In this case, post hoc analysis revealed a lower basal number of BrdU^+^/SOX2^+^ cells in MP Controls compared with BP Controls, indicating that monoparental rearing reduces baseline proliferation of NSC in the SGZ (Figure 2B). As with total BrdU^+^ cells, cohabitation increased BrdU^+^/SOX2^+^ cells in the BP Cohabitation group compared with BP Controls, whereas no significant change was observed in MP Cohabitation males relative to MP Controls. A comparison between BP and MP Cohabitation groups showed a higher number of BrdU^+^/SOX2^+^ cells in BP Cohabitation males (Figure 2B).

These findings are consistent with the significant effect of rearing (F_(1,16)_ = 23.979, *p* < 0.001) and treatment (F_(1,16)_ = 7.287, *p* = 0.016) on the percentage of proliferating NSCs (BrdU^+^/SOX2^+^, Figure 2C). Fisher LSD post hoc test showed a higher percentage of proliferating NSCs in the BP Control in comparison to the MP Control (*p* = 0.028, white bars in Figure 2C), an increase in BP Cohabitation vs. BP Control (*p* = 0.009, Figure 2C), and higher percentages in BP Cohabitation vs. MP Cohabitation. No significant differences were found between the MP Cohabitation and MP Control groups, nor in the number of SOX2^+^ cells per mm^2^ [rearing (F_(1,16)_ = 0.477, *p* = 0.50), and treatment (F_(1,16)_ = 0.518, *p* = 0.482)].

Overall, these results suggest that rearing conditions influence basal NSC proliferation in both neurogenic niches, with reduced proliferation rates observed in MP males. In BP males, cohabitation for 24 h increased proliferative activity in both neurogenic niches, similar to effects previously reported after 48 h [27]. In contrast, MP males showed increased proliferating NSCs only in the SVZ. The blunted response in SGZ after cohabitation may be related to the delayed pair-bond formation previously observed in MP voles.

#### 2.3.3. Cell Proliferation Levels in the SVZ of Female Prairie Voles

**Figure 3:**
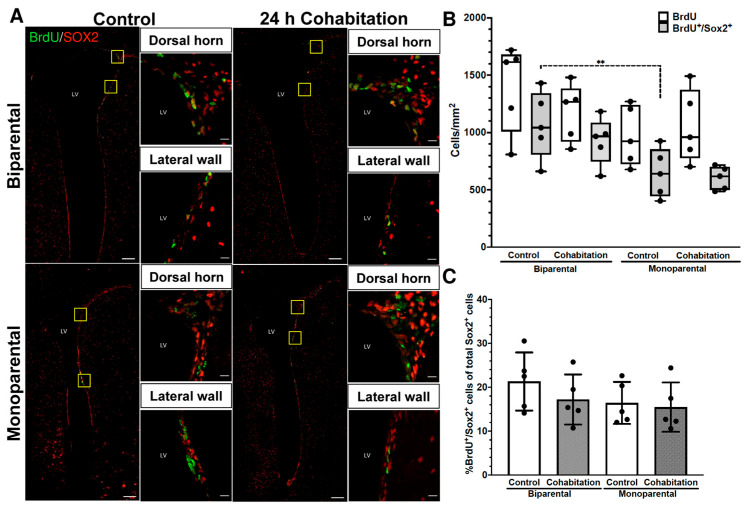
Cell proliferation levels in the SVZ of female prairie voles. (**A**) Representative photomicrographs of BrdU^+^/SOX2^+^ cells in the SVZ of BP and MP females. Scale bar in panoramic image, 100 μm; insets 10 μm. (**B**) Quantification of BrdU^+^ and BrdU^+^/SOX2^+^ cells per mm^2^. (**C**) Percentage of BrdU^+^/SOX2^+^ cells from total SOX2^+^ cells. Significance level ** *p* < 0.01.

Representative photomicrographs of BrdU^+^/SOX2^+^ cells in females are shown in Figure 3A. No significant differences were found in the number of BrdU^+^ cells per mm^2^ in the SVZ (H_(3)_ = 4.577, *p* = 0.206; Figure 3B). However, significant differences were detected in the number of BrdU^+^/SOX2^+^ cells per mm^2^ (H_(3)_ = 9.514, *p* = 0.023). Under basal conditions BP Control females showed a higher number of BrdU^+^/SOX2^+^ cells than MP Controls (n = 5, *p* = 0.032; dashed line Figure 3B).

No significant effect of rearing of treatment were found on the percentage of BrdU^+^/SOX2^+^ cells [rearing (F_(1,16)_ = 1.676, *p* = 0.21); treatment (F_(1,16)_ = 0.973, *p* = 0.339)] or on the number of SOX2^+^ cells per mm^2^ [rearing (F_(1,16)_ = 7.128, *p* = 0.075); treatment (F_(1,16)_ = 0.370, *p* = 0.551)].

#### 2.3.4. Cell Proliferation Levels in the SGZ of Female Prairie Voles

**Figure 4:**
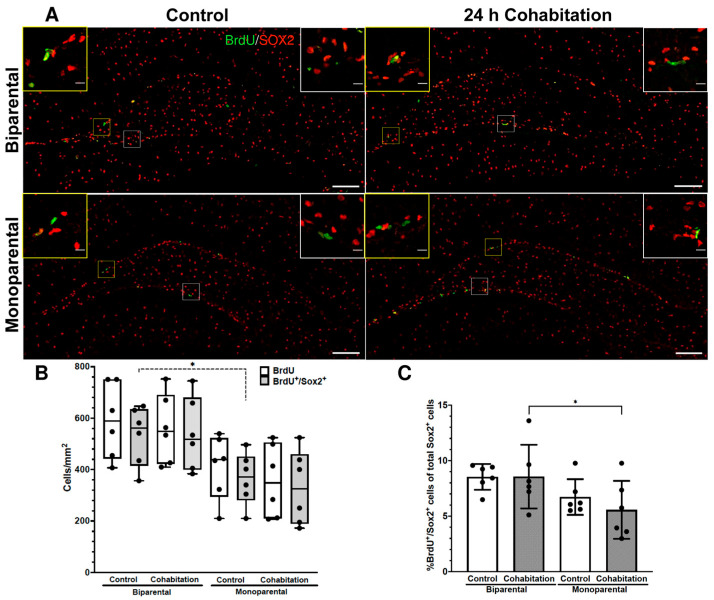
Cell proliferation levels in the SGZ of female prairie voles. (**A**) Representative photomicrographs of BrdU^+^/SOX2^+^ cells in BP and MP females. Yellow insets showed cells in the suprapyramidal blade, white insets in the infrapyramidal blade. Scale bar in panoramic image 100 μm; inserts 10 μm. (**B**) Quantification of BrdU^+^ and BrdU^+^/SOX2^+^ cells per mm^2^. (**C**) Percentage of BrdU^+^/SOX2^+^ cells from total SOX2^+^ cells. Significance levels * *p* < 0.05.

Similar results were obtained in the SGZ (Figure 4A). No significant differences were found in the number of BrdU^+^ cells per mm^2^ (H_(3)_ = 7.78, *p* = 0.051; Figure 4A,B). In contrast, significant differences were observed in the number of BrdU^+^/SOX2^+^ cells per mm^2^ (H_(3)_ = 8.713, *p* = 0.033; Figure 4A,B). Post hoc analysis revealed a higher number of BrdU^+^/SOX2^+^ cells in the BP Controls than in MP Controls (dashed line in Figure 4B).

For the percentage of total proliferating NSCs, we found a significant effect of rearing (F_(1,20)_ = 7.282, *p* = 0.014) but not of treatment (F_(1,20)_ = 0.403, *p* = 0.533). Post hoc analysis indicated a significantly higher percentage of BrdU^+^/SOX2^+^ cells in BP Cohabitation vs. MP Cohabitation females (*p* = 0.027). Similarly to the SVZ, no significant differences were found in the number of SOX2^+^ cells per mm^2^ [rearing (F_(1,16)_ = 6.199, *p* = 0.060); treatment (F_(1,16)_ = 0.497, *p* = 0.489)].

In summary, these results suggest that rearing influences NSCs proliferation in both neurogenic niches of females, with reduced basal proliferative rates in MP animals. Notably, we did not observe an effect of 24 h of cohabitation on NSCs proliferation in either MP or BP females. To characterize the natural response of female neurogenic niches at the onset of cohabitation, we administered BrdU during the first 4 h to label mitotic cells in non–hormone-treated females. Our results show high variability in the number of newborn cells in the SVZ and the SGZ, which could be explained by a differential response of females to the initial male exposure.

#### 2.3.5. Sexual Dimorphisms in the Proliferative Activity of the Neurogenic Niches

To examine sex differences, we compared the numbers of BrdU^+^ and BrdU^+^/SOX2^+^ cells between males and females (Table 3).

Under basal conditions, BP females had more BrdU^+^ and BrdU^+^/SOX2^+^ cells in the SVZ than BP males, and MP females had more than MP males. In the Cohabitation condition, BP and MP males had more BrdU^+^/SOX2^+^ cells in the SVZ than BP and MP females, respectively.

In the SGZ, BP males had more BrdU^+^ and BrdU^+^/SOX2^+^ cells than females under basal and cohabitation conditions. MP males in the Cohabitation group also had more BrdU^+^ cells than MP females.

### 2.4. Enrichment of Epigenetic Marks in Newborn Cells

H3K4me3 is a promoter-associated mark enriched near transcription start sites and is generally linked to transcriptional competence and increased promoter activity [43], whereas H3K27me3 is classically associated with transcriptional repression [44]. Our results indicate that MP voles exhibit lower cell proliferation than BP voles. To explore whether this reduction was accompanied by differences in the enrichment of epigenetic marks in newborn cells (BrdU^+^ cells), we quantified the fluorescence intensity of H3K4me3 and H3K27me3. H3K4me3 and H3K27me3 signal intensities can vary substantially across cells and may also be influenced by section thickness, nuclear plane of section, and thresholding parameter during image acquisition and quantification. In our material, all BrdU^+^ nuclei exhibited a detectable H3K4me3 and H3K27me3 signal; however, the intensity was heterogeneous, which may create the appearance of “negative” cells under conservative visualization settings.

#### 2.4.1. H3K4me3 Fluorescence Intensity in BrdU^+^ Cells of the Male SVZ

**Figure 5:**
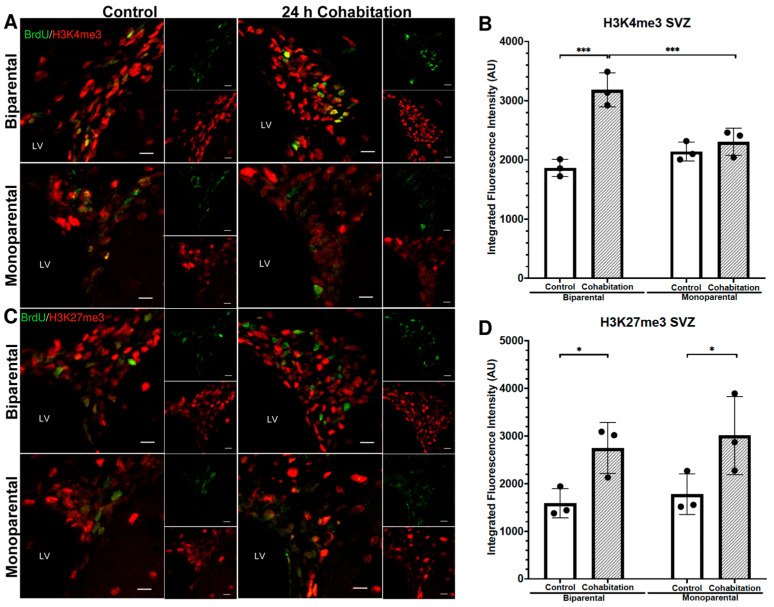
Epigenetic marks enrichment (H3K4me3 and H3K27me3) in the BrdU^+^ cells residing in the SVZ of male prairie voles. (**A**) Representative photomicrographs of BrdU^+^/H3K4me3^+^ cells. Scale bar: 10 μm. (**B**) Quantification of H3K4me3 integrated fluorescence intensity in BrdU^+^ cells. (**C**) Representative BrdU^+^/H3K27me3^+^ photomicrographs. Scale bar: 10 μm. (**D**) Quantification of H3K27me3 integrated fluorescence intensity. Significance levels * *p* < 0.05 and *** *p* < 0.001.

Analysis of H3K4me3 fluorescence intensity (integrated density) in BrdU^+^ cells revealed significant effects of rearing (F_(1,8)_ = 6.01, *p* = 0.04) and treatment (F_(1,8)_ = 36.57, *p* < 0.001, Figure 5A,B). Post hoc analysis revealed that BP Cohabitation males exhibited higher levels of H3K4me3 than BP Controls and MP Cohabitation males. No significant differences were found between MP Control and MP Cohabitation groups. The increased activation mark in the BP Cohabitation group may partially account for the higher proliferation levels observed.

#### 2.4.2. H3K27me3 Fluorescence Intensity in the BrdU^+^ Cells of the Male SVZ

For H3K27me3 fluorescence (integrated density) in BrdU^+^ cells of the SVZ, we found a significant effect of treatment (F_(1,8)_ = 13.92, *p* = 0.006), but not of rearing (F_(1,8)_ = 0.508, *p* = 0.49; Figure 5 C,D). Post hoc analysis showed that both BP and MP Cohabitation males exhibited higher H3K27me3 levels than their respective controls. No significant differences were detected between MP and BP males after cohabitation with a female.

#### 2.4.3. H3K4me3 Fluorescence Intensity in BrdU^+^ Cells of the Male SGZ

**Figure 6:**
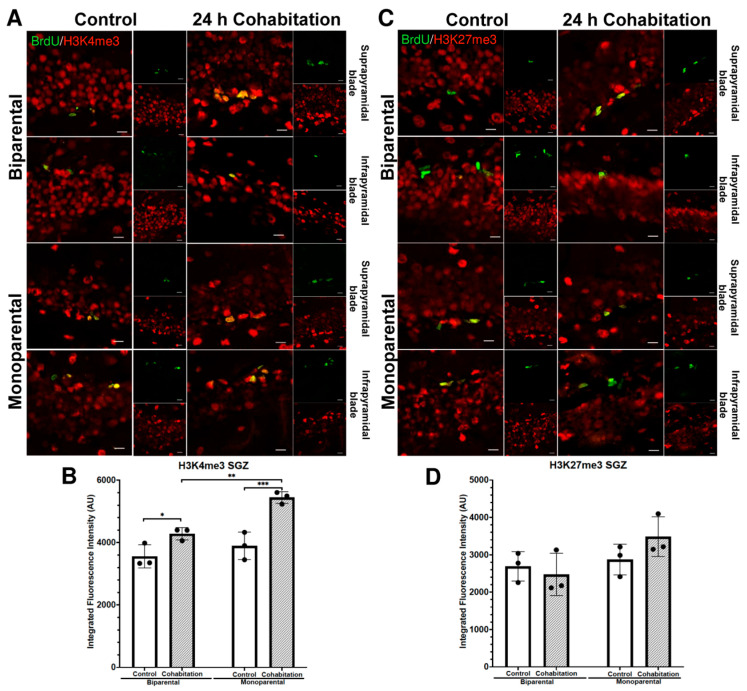
Epigenetic marks enrichment (H3K4me3 and H3K27me3) in the BrdU^+^ cells residing in the SGZ of male prairie voles. (**A**) BrdU^+^/H3K4me3^+^ cells in the suprapyramidal and infrapyramidal blades of BP (top) and MP (bottom) males. Scale bar: 10 μm (**B**) Quantification of H3K4me3 fluorescence intensity. (**C**) BrdU^+^/H3K27me3^+^ cells in the suprapyramidal and infrapyramidal blades. Scale bar: 10 μm. (**D**) Quantification of H3K27me3 fluorescence intensity. Significance level: * *p* < 0.05, ** *p* < 0.01 and *** *p* < 0.001.

In the SGZ, H3K4me3 fluorescence intensity (integrated density) in BrdU^+^ cells was significant affected by both rearing (F_(1,8)_ = 16.58, *p* = 0.004) and treatment (F_(1,8)_ = 38.45, *p* < 0.001; Figure 6A,B). Post hoc analysis showed that Cohabitation males had higher H3K4me3 levels than Controls in both BP and MP groups. Interestingly, H3K4me3 levels were lower in BP Cohabitation males than in MP Cohabitation group.

#### 2.4.4. H3K27me3 Fluorescence Intensity in BrdU^+^ Cells of the Male SGZ

For H3K27me3 fluorescence intensity in BrdU^+^ cells of the SGZ, no significant effects of rearing (F_(1,8)_ = 4.59, *p* = 0.064) or treatment (F_(1,8)_ = 0.514, *p* = 0.494; Figure 6C,D) were detected.

#### 2.4.5. H3K4me3 Fluorescence Intensity in BrdU^+^ Cells of the Female SVZ

**Figure 7:**
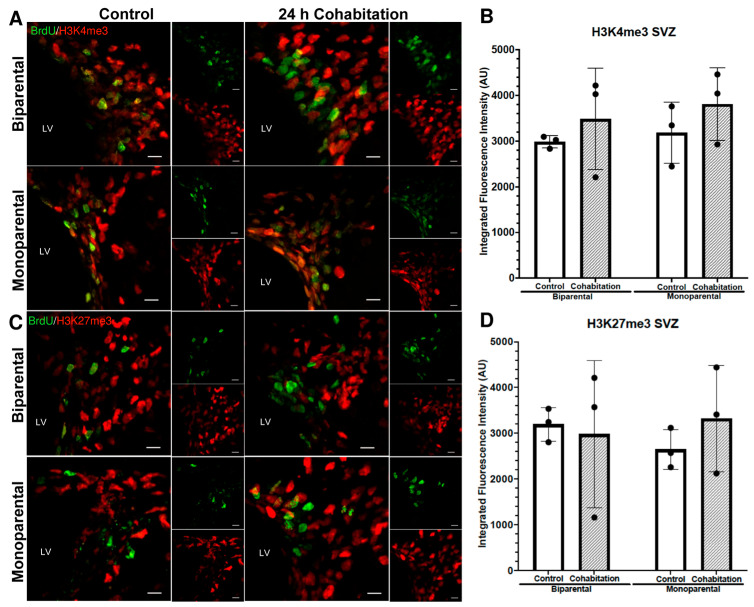
Epigenetic marks enrichment (H3K4me3 and H3K27me3) in the BrdU^+^ cells residing in the SVZ of female prairie voles. (**A**) BrdU^+^/H3K4me3^+^ cells in BP (top) and MP (bottom) females. Scale bar: 10 μm. (**B**) Quantification of H3K4me3 fluorescence intensity. (**C**) BrdU^+^/H3K27me3^+^ cells of BP (top) and MP (bottom) females. Scale bar: 10 μm. (**D**) Quantification of H3K27me3 fluorescence intensity.

In females, H3K4me3 fluorescence intensity (integrated density) of H3K4me3 in BrdU^+^ cells of SVZ show no significant effect of rearing (F_(1,8)_ = 0.354, *p* = 0.568) or treatment (F_(1,8)_ = 1.636, *p* = 0.237, Figure 7A,B).

#### 2.4.6. H3K27me3 Fluorescence Intensity in BrdU^+^ Cells of the Female SVZ

Similarly, H3K4me3 fluorescence intensity in BrdU^+^ cells of SVZ did not differ significantly between BP and MP females (F_(1,8)_ = 0.0289, *p* = 0.869), nor between Control and Cohabitation groups (F_(1,8)_ = 0.150, *p* = 0.709, Figure 7C,D).

#### 2.4.7. H3K4me3 Fluorescence Intensity in BrdU^+^ Cells of the Female SGZ

**Figure 8:**
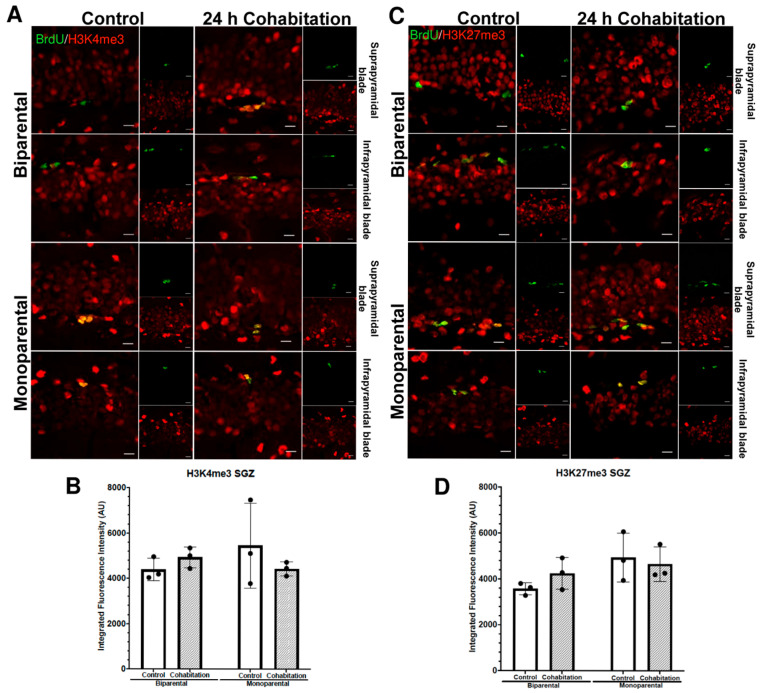
Epigenetic marks enrichment (H3K4me3 and H3K27me3) in the BrdU^+^ cells residing in the SGZ of female prairie voles. (**A**) BrdU^+^/H3K4me3^+^ cells in suprapyramidal and infrapyramidal blades of BP (top) and MP (bottom) females. Scale bar: 10 μm. (**B**) Quantification of H3K4me3 intensity. (**C**) BrdU^+^/H3K27me3^+^ cells in BP (top) and MP (bottom) females. Scale bar: 10 μm. (**D**) Quantification of H3K27me3 intensity.

In the SGZ of females, H3K4me3 fluorescence intensity in BrdU^+^ cells showed no significant effect of rearing (F_(1,8)_ = 0.2294, *p* = 0.644) or treatment (F_(1,8)_ = 0.1780, *p* = 0.684, Figure 8A,B).

#### 2.4.8. H3K27me3 Fluorescence Intensity in BrdU^+^ Cells of the Female SGZ

Finally, H3K4me3 fluorescence intensity in BrdU^+^ cells of females SVZ did not differ significantly between BP and MP females (F_(1,8)_ = 4.23, *p* = 0.074) or between Control and Cohabitation groups (F_(1,8)_ = 0.203, *p* = 0.664, Figure 8C,D).

## 3. Discussion

### 3.1. Monoparental Rearing Alters Basal Proliferation of Adult NSCs in the SVZ and SGZ

Parental deprivation exerts detrimental effects on adult neurogenesis in both monoparental and biparental species. In rats, maternal separation significantly decreases the number of newborn cells in the SGZ in males [45] and reduces the granule cell population in females [46]. In mice, maternal separation reduces the number of adult newborn neurons that integrate into the dentate gyrus (DG), without altering overall cell proliferation [47]. In *Microtus mandarinus*, a biparental species, He and colleagues employed paternal deprivation during postnatal day 14–21 as a model of emotional attachment disruption. They reported fewer newborn cells and neurons in the DG of MP females compared with BP females, while no differences were observed in males; however, the number of immature neurons decreased in MP voles of both sexes [31].

In the present study, we show that the absence of the father during rearing reduces NSCs proliferative activity in the SVZ and SGZ of adult prairie voles, resulting in significantly fewer proliferating cells in MP males and females compared with BP animals. This suggests an altered basal activation of NSCs without affecting the total NSC population, since we did not find significant differences in the population of SOX2 expressing cells, a NSCs marker. Nevertheless, because animals were euthanized 24 h after the behavioral manipulation, we cannot exclude the possibility that a subset of BrdU^+^ cells had already progressed toward transit-amplifying and neuronal-commitment stages (e.g., T-Box Brain Protein 2/Eomesodermin (TBR2/EOMES), Minichromosome Maintenance Complex Component 2 (MCM2), Neuronal Differentiation 1 (NEUROD1)) and/or early-migrating neuroblasts (Doublecortin; DCX). Finally, although BrdU administration protocols and experimental design differ between the He et al. study and ours, MP voles consistently showed fewer adult newborn cells than BP voles.

Paternal deprivation in monogamous species constitutes an early-life stressor with long-lasting effects into adulthood [4,5,6]. MP prairie voles of both sexes express higher levels of brain derived neurotrophic factor (BDNF) and its receptor tropomyosin receptor kinase B (TrkB) in the hippocampus [48]. TrkB is critical for neuronal survival, cell proliferation, development, synaptic plasticity, memory, and cognition [49].

BDNF, a neurotrophic factor known to enhance adult neurogenesis; may exert distinct effects in the SVZ and SGZ [50,51]. *In vitro,* BDNF treatment of SGZ-derived NSCs induces cell proliferation, neuronal differentiation, and survival [52]. Similarly, BDNF increases the number and size of neurospheres obtained from SVZ NSCs of male and female voles that cohabit with opposite-sex animals, and in females it favors neuronal differentiation [28], whereas intrahippocampal BDNF infusion increases the number of newborn neurons [53]. In the SVZ-OB system, genetic disruption of TrkB signaling reduces the number of newborn neurons without necessarily altering NSC proliferation [54,55]. Collectively, these findings are consistent with a predominant role for BDNF/TrkB in survival and/or maturation rather than in the earliest proliferative step. Accordingly, future studies should test whether elevated BDNF/TrkB signaling in MP animals partially compensates for reduced proliferation at later stages of the neurogenic process (e.g., ~45 days post-labeling).

With respect to glucocorticoid signaling, Tabbaa et al. reported no differences in hippocampal glucocorticoid receptor (GR) α protein in males, but increased GR β in females following paternal deprivation [48]. In a related monogamous vole model, female mandarin voles (but not males), are particularly sensitive to paternal deprivation, displaying lower levels of GR and BDNF in the DG, and higher corticosterone and adrenocorticotropin serum levels [56]. These studies support the idea that in addition to behavioral alterations, MP rearing disrupts the hypothalamic–pituitary–adrenal axis and several neurochemical systems that may influence the adult neurogenesis process. While, these sex-dependent patterns do not straightforwardly account for the reduced proliferation observed in both sexes in our study, they underscore the importance of explicitly modeling sex as a biological variable in mechanistic follow-up work.

In this regard, high glucocorticoids levels linked to stress have a deleterious effect on adult neurogenesis [57,58]. Tanapat and colleagues demonstrated that exposure to a predator odorant reduces DG cell proliferation in male rats, an effect prevented when the glucocorticoid surge is blocked [59]. Moreover, exogenous corticosterone administration decreases SGZ cell proliferation and the survival of DG granule neurons with subtle sex differences [60]. The hippocampus is of particular interest because of its role in cognition and stress responses and its abundant expression of GRs in neurons [61] and progenitor cells [62], especially when compared to the OB [63,64]. However, corticosterone treatment also reduces the number of newborn cells in the SVZ [65], and chronic stress decreases the population of BrdU^+^ cells, neuroblasts, and newborn neurons in the SVZ-OB system [66]. Future studies should quantify GR pathway components and downstream transcriptional targets in a sex-stratified manner.

Oxytocin is a neuropeptide primarily synthesized in magnocellular neurons of the paraventricular and supraoptic nuclei of the hypothalamus [67]. In prairie voles, this neuropeptide has been identified as a crucial participant in pair-bonding behavior [68,69], parental care [70], and lactation [71], although recent evidence indicates that these processes can occur in oxytocin receptor null voles [72]. Interestingly, oxytocin promotes NSCs proliferation in the rat hippocampus and counteracts the deleterious effect of stress hormones [73,74]. *In vitro,* oxytocin increases the proliferation of SVZ neurospheres obtained from prairie voles that cohabit with opposite-sex animals [28]. Given that oxytocin receptors are sparsely expressed by DG NSCs and mature granule neurons, it has been proposed that oxytocin enhances adult neurogenesis through a non-cell autonomous mechanism mediated by excitatory inputs from CA3 pyramidal neurons to the DG [75].

In summary, MP rearing induces neurochemical and neuroendocrine alterations that may impact adult neurogenesis. Further investigations considering sex-dimorphic differences are required to clarify the extent to which disruptions in these systems contribute to the reduced proliferation observed in MP prairie voles.

### 3.2. Monoparentally Reared Male Voles Show Lower Levels of NSCs Proliferation After 24 h of Cohabitation with a Mating Partner

As mentioned before, several studies support the involvement of the OB and DG in processing sensory information associated with socio-sexual incentives, particularly in pair-bond formation, which may in turn stimulate the associated neurogenic niches. Consistent with this idea, previous work from our group showed that 48 h of cohabitation with mating increases cell proliferation and neuronal differentiation in BP male voles [27,28]. Here, we show a similar effect after 24 h, with an increase in NSCs proliferation in both the SVZ and SGZ of males.

Although to a lesser extent, MP prairie voles that cohabitate with an opposite-sex partner also exhibit higher NSCs proliferation in the SVZ compared with MP controls, suggesting that this niche remains responsive to socio-sexual stimulation. However, unlike BP males, MP males did not show a significant increase in BrdU^+^ cells in the SGZ following cohabitation. This result highlights a deficient response of the hippocampal neurogenic niche in MP voles, which becomes particularly evident under challenging conditions.

Notably, He and colleagues reported that MP mandarin voles exhibit deficits in a social recognition test, suggesting functional impairment in hippocampus-dependent processes [31]; whereas MP prairie voles show normal performance in olfaction-related tasks [6]. We speculate that reduced NSCs proliferation in the DG may ultimately diminish the population of newborn neurons required for discriminating similar experiences and forming new memories [76,77,78]. Interestingly, MP voles also exhibit delayed pair-bond formation, a phenotype that further suggests disruptions in hippocampal plasticity mechanisms.

Our current dataset does not allow us to determine whether the increase in the number of BrdU/SOX2^+^ cells observed in voles cohabiting with a female would reverse after returning animals to control housing conditions. Nevertheless, there are biologically plausible reasons to anticipate partial normalization over time, as well as reasons to consider that some effects may persist. First, survival of newly generated cells is tightly regulated during an early post-mitotic window; a substantial fraction of newborn cells is eliminated, and survival and functional integration are strongly influenced by experience and physiological state (e.g., learning, physical activity, and socio-sexual stimulation) [79,80,81]. Second, once pair-bonding has been established, separation from the partner can elicit a stress-like state in both members of the pair [82], which may further modulate cell survival and/or proliferation depending in a time- and context-dependent manner.

### 3.3. Proliferative Levels of Adult NSCs in Female Voles Do Not Change After 24 h of Cohabitation

In the present study, we did not detect changes in NSCs proliferation after 24 h of cohabitation in either BP or MP females. These findings may reflect differences in the temporal dynamics of the neuroendocrine response to male sensory stimuli in females.

Socially naive female prairie voles remain anestrous until exposure to male sensory cues, followed by an increase in their ovarian hormonal levels leading to estrous induction [83]. In a study by Cohen-Parsons and Carter, serum estradiol levels and nuclear estrogen receptor binding in the brain significantly increased after 18 h of continuous male exposure, whereas no significant differences were detected at 6 h [84]. Progesterone levels increase approximately 72 h after mating [85].

Cell proliferation induced by a socio-sexual environment in females appears to have a strong hormonal component, as prairie voles induced into estrous by male exposure for 48 h or ovariectomized females treated with estradiol show a significant increase in BrdU^+^ cells in the SVZ-RMS and SGZ [26,30]. Rodent NSCs express estrogen [86] and progesterone [87] receptors, and their proliferation is stimulated by these hormones [87,88]. Peripheral responses to estradiol are activated at much lower serum estradiol concentrations. In contrast, higher serum estradiol accumulation is required to initiate a reaction in the central nervous system [89]. In addition, the increased levels of estradiol in the central nervous system might have an effect by an indirect pathway, potentially involving activation of estrogen receptors in astrocytes with the subsequent engagement of a BDNF dependent signaling pathway that induces proliferation in the neurogenic niche [90]. Low doses of estradiol (0.5 μM) in cell culture promote SVZ NSCs proliferation in female voles that cohabit with males, and decrease neuronal differentiation at 1 μM. Progesterone modulates differentiation, but there is no neurite formation in males and females that cohabit with opposite-sex voles [28].

On the other hand, our partner preference data show that most BP females and a subset of MP females, already preferred their sexual partner over a novel male, suggesting that early pair-bond formation in females does not necessarily parallel early changes in NSCs proliferation. It is likely that a longer cohabitation period and/or the progression of the endocrine response are required before detectable changes occur in female neurogenic niches.

### 3.4. Monoparental Rearing Modifies the Epigenetic Profile of Adult Newborn Cells in the SVZ and the SGZ

Given our study design (baseline and a single post-stimulation timepoint at 24 h), we cannot directly determine whether the observed epigenetic differences are transient, persistent, or permanent. Epigenetic regulation operate across multiple temporal scales: rapid enzyme recruitment and chromatin-associated regulatory cycles have been reported on the order of minutes to hours in cellular systems [91,92], whereas other epigenetic alterations—particularly those linked to early-life experience—can persist long term and correlate with stable phenotypes in adulthood. Consistent with the latter, experience-dependent epigenetic mechanisms have been implicated in enduring behavioral and neurobiological outcomes. Parental care exerts long-lasting effects on offspring, including stable epigenetic modifications. For example, female rats that receive high levels of licking/grooming during postnatal development provide higher levels of maternal licking/grooming to their own pups at adulthood and variations in maternal care has been linked to differential DNA methylation at the glucocorticoid receptor gene promoter, correlating with reduced receptor expression in adulthood [93]. In monogamous voles, paternal deprivation has similarly been associated with enduring behavioral differences and altered DNA methylation at candidate loci such as the vasopressin receptor 1a gene [94]. In addition, Tabbaa and colleagues demonstrated that MP raised voles have higher levels of total histone 3 (H3 total) and the ratio of histone 3 acetylation to H3 total, a process that generally activates gene transcription and synaptic plasticity in the hippocampus through epigenetic mechanisms [48]. Maternal separation also alters *in vitro* neuronal differentiation of rat NSCs via methylation of the retinoic acid receptor (RAR) promoter [95]. Taken together, these findings support the hypothesis that paternal deprivation may induce epigenetic changes that persist into adulthood and may be detectable in adult neural stem/progenitor populations, potentially influencing daughter cells.

Therefore, we evaluated enrichment of two epigenetic marks in newborn cells of adult neurogenic niches: H3K4me3, related to transcriptional activation [43] and H3K27me3, associated with gene silencing [96]. Our results showed a significant increase in both H3K4me3 and H3K27me3 in newborn cells of the SVZ of BP prairie voles after 24 h of cohabitation, whereas only H3K27me3 increased in SVZ BrdU^+^ cells of MP prairie voles. In the SGZ, BP and MP voles showed higher levels of H3K4me3 after cohabitation, although the increase was significantly greater in MP voles. However, no significant differences were detected in H3K27me3 levels in the SGZ. Consistent with the NSCs proliferation data, no significant differences in these epigenetic marks were observed in females.

Epigenetic mechanisms stabilize cell type-specific gene expression patterns to guarantee lineage commitment [97]. The combination of different histone N-terminal modifications, such as acetylation, methylation, and phosphorylation known as the “histone code” determines chromatin accessibility to the transcriptional machinery [98]. NSCs quiescence and activation depend on multiple mechanisms; dynamic changes in DNA and histone methylation patterns guide these cells and their progeny through critical fate decisions, including self-renewal, differentiation and maturation [34,99,100].

The main differences between rearing systems (BP vs. MP) were that H3K4me3 levels in SVZ newborn cells of MP males did not change after 24 h of cohabitation; whereas levels of this mark were higher in SGZ newborn cells of MP males compared to BP males. These alterations in the histone code of NSCs in MP voles may lead to changes in gene expression patterns, thereby contributing to the differences in cell proliferation. As mentioned, 48 h of cohabitation with mating promotes cell proliferation and neuronal differentiation in BP prairie voles, suggesting changes in gene expression within neurogenic cell cohorts. Using bulk RNA-seq analysis of adult neurogenic niches, we previously observed differential gene expression in cohabitation vs. social isolation, depending on the sex and neurogenic niche. In females, cohabitation with mating identified only eight differentially expressed genes (DEGs), whereas in males, the number ranged from 37 to 161 DEGs. Gene ontology analysis showed that in the SVZ, downregulated genes were related to cell adhesion (Muc5b, Robo3, Muc2) in females, and to nuclear division (Zcwpw1, Rnf212b, L3mbtl1, Tdrd12) in males; whereas upregulated genes were related to neuropeptide hormone activity (Vip, Hcrt) in females, and to ion channel complex (Kcne1l, Catsper2, Best3) in males. In the DG, downregulated genes included the GR and predicted glucocorticoid receptor targets (Pla2g4b, Cldn20, Fmo1, Robo3) in females; and genes related to neuroblast polarization (Dpy19l2, Casc1, Spata46) and extracellular matrix (Col5a2, Sfrp2, Col16a1, Rxfp1, Mia, Nphs1, Gas2) in males [101]. These results reveal sex-specific molecular responses to cohabitation in neurogenic niches. Females show molecular signatures of neuropeptide activation in the SVZ and reduced GR signaling in the DG, suggesting a neuroendocrine modulation that may support adaptive neurogenic responses to pair-bonding. Males show signatures of cell-cycle suppression in the SVZ and structural and developmental programs in the DG, which may be necessary for proliferation and neuroblast maturation. Further research is needed to examine gene expression differences in MP prairie voles.

## 4. Materials and Methods

### 4.1. Animals

Male and female prairie voles were raised at the Animal Facility of the Instituto de Neurobiologia of the Universidad Nacional Autonoma de Mexico (Queretaro, Mexico). The colony founders were kindly provided by Dr. Larry J. Young (Emory University, Atlanta, GA, USA). All animals were housed in 40 × 20 × 20 cm acrylic cages with pine chip bedding and recycled paper. Subjects were maintained on a 14 h light/10 h dark cycle at 20–25 °C, with *ad libitum* access to water and food (rabbit diet high fiber 5326, oats and sunflower seeds; LABDIET, St. Louis, MO, USA). All experimental procedures were approved by the Animal Care Committee of the Instituto de Neurobiologia.

To obtain prairie voles raised by the mother (MP family), we removed the male from the housing cage 18 days after the onset of cohabitation with the female. This ensured pups viability [4] and the absence of the father at birth. MP and BP voles were weaned 21 days after birth.

Once all subjects reached 3 months of age, they were randomly assigned to two experimental conditions: (a) Control, sexually naive male and female voles that, during the test, were kept in their housing cage with same-sex, same-family-condition conspecific and (b) Cohabitation, males and females that cohabitated for 24 h with an unrelated opposite-sex vole. Although epigenetic remodeling can occur across diverse timescales, there is precedent for relatively rapid chromatin-enzyme dynamics associated with transcriptional regulation. For example, methylation-related regulatory cycles have been reported on the order of hours in cellular systems [91,92]. We therefore selected 24 h to assess neural stem/progenitor proliferation, given that the cell-cycle length of SVZ B1 cells has been estimated at ~18 h [102]. Accordingly, the 24 h interval provides a practical window to capture most BrdU-labeled cells within neurogenic niches prior to subsequent migration and differentiation [102].

All experimental animals remained gonadally intact. Stimuli females (partners and strangers) were ovariectomized and hormonally primed with estradiol benzoate (0.5 μg/vole daily; E8515 Sigma-Aldrich, St. Louis, MO, USA) for four days to induce sexual receptivity [103,104,105]. Stimuli males (partners and strangers) were vasectomized. At the beginning of the experiment, Control and Cohabitation groups were administered the mitotic marker 5-Bromo-2′-deoxyuridine, BrdU (B5002, Sigma-Aldrich, St. Louis, MO, USA) at 3 doses of 100 mg/kg, one dose every two hours.

### 4.2. Sexual Behavior

Sexual behavior was recorded during the first 6 h of cohabitation. During the first hour, the number and latency of mounts, intromissions, and ejaculations in males and lordosis in females, were registered.

### 4.3. Partner Preference Test

After 24 h of cohabitation, experimental subjects underwent the partner preference test, PPT (described in [6]). Briefly, the test started by placing the experimental subject in a central compartment of a transparent acrylic box (30 × 40 × 30 cm). The lateral compartments (10 × 10 × 10 cm) housed, on one side, the sexual partner and on the other, an opposite-sex stranger that was not family related to the experimental vole (i.e., not a cousin, sibling, father, or mother). The partner and stranger voles were confined to their compartment. The central compartment allows the experimental animal to avoid contact with both stimuli voles.

During the test, a video recording was made to register the number of entries into both compartments, the time spent with each animal and huddling events (a parameter of pair-bond formation). The test lasted three hours, and the first two hours were manually analyzed. To determine preference for the partner or the stranger, a preference index (PI) was calculated by dividing the time spent with the partner by the sum of the time spent with the partner and the stranger.

### 4.4. Tissue Preparation and Immunofluorescence

All experimental subjects were sacrificed immediately after PPT completion. Once anesthetized with an overdose of pentobarbital (6.3 mg per vole, Q-7048-044, Cheminova, Mexico), intracardiac perfusion was performed with 0.1 M PBS followed by 4% paraformaldehyde (158127, Sigma-Aldrich, St. Louis, MO, USA). Brains were collected and cryopreserved in 30% sucrose (4072-01, J.T. Baker, Phillipsburg, NJ, USA). Subsequently, 30 μm coronal sections were cut in a cryostat and stored in antifreeze solution until immunostaining. To determine the number of proliferating NSCs, we used an anti-BrdU antibody (1:800, rat monoclonal antibody, OBT0030, AbD Serotec, Hercules, CA, USA) in combination with an anti-SOX2 antibody (1:1000, rabbit monoclonal antibody, ab92494, abcam, Waltham, MA, USA). Epigenetic marks in proliferating cells of neurogenic niches were detected using the following antibodies: H3K4me3 (1:3000, rabbit polyclonal antibody, ab8580, abcam, Waltham, MA, USA) and H3K27me3 (1:3000, rabbit polyclonal antibody, ABE44, Sigma-Aldrich, St. Louis, MO, USA), using a standard immunochemistry protocol. Briefly, four slices per brain were selected for analysis of each immunophenotypical marker. Brain slices were rinsed three times with 0.1 M TBS (pH 7.6), followed by incubation in 0.1% Triton X-100. For BrdU detection, slices were incubated in 2 N HCl at 37 °C for 30 min and washed three times with TBS. After blocking in 10% donkey serum (D9663, Sigma-Aldrich, St. Louis, MO, USA)/0.1% TX-100, slices were incubated overnight at 4 °C with the corresponding primary antibody in a solution containing 1% donkey serum/ 0.1% TX-100. After three rinses in TBS, slices were incubated for 2 h at room temperature with the appropriate secondary antibody [1:3000 goat Alexa Fluor 488 anti-rat (A11006) or 568 anti-rabbit (A11036), Thermo Fisher Scientific, Waltham, MA, USA] in a solution containing 2% donkey serum/ 2% TX-100. Finally, brain slices were rinsed with TBS and mounted on glass slides with Aqua-Poly/Mount (18606-5, Polysciences, Inc., Warrington, PA, USA) mounting medium.

### 4.5. Microscopy and Image Analysis

Photomicrographs were obtained from four brain sections and both hemispheres were analyzed per subject (n = 5 or 6 per group). Images were acquired with a Zeiss confocal microscope (AX10, Zeiss, Graz, Austria) equipped with ZEN 2012 software (version 14.0.29.201). For cell counting, photomicrographs were acquired with a 20× objective at 10 different Z-planes; for co-expression analysis, z-stack images were acquired with a 63× objective. All images were analyzed using Fiji (Image J version 2.16.0) and results expressed as mean ± SDM (cells/mm^2^) or as the percentage (mean ± SDM) of double-positive cells relative to the total number of SOX2^+^ cells, when applicable. For H3K4me3 and H3K27me3 fluorescence intensity (integrated density) measurements, we analyzed 63× Z-stack images of around 30 individual BrdU^+^ cells per subject and epigenetic marker (n = 3 per group). After obtaining z-projections, BrdU^+^ cells were identified and images were converted to grayscale. Integrated density was measured in each individual nucleus using manual regions of interest (ROIs) and background signal was subtracted. Results are represented as integrated fluorescence intensity in arbitrary units (mean ± SDM).

### 4.6. Statistical Analysis

Statistical analyses were performed using SigmaPlot (version 11.0) or GraphPad Prism (version 10.5.0). A Two-Way ANOVA was employed for comparisons between groups, except when data failed normality (Shapiro–Wilk) and equal variance tests, in which case a Kruskal–Wallis Analysis of Variance on Ranks was applied. Fisher’s LSD multiple comparisons test or the Mann–Whitney U test was used to identify statistically significant differences. T-student test was applied for comparisons between two independent groups. A *p* value less than 0.05 was considered statistically significant.

## 5. Conclusions

Paternal deprivation and maternal separation exert behavioral, neurochemical, and neuroendocrine effects that persist into adulthood. The study was designed as an initial screening analysis to detect early differences associated with parenting conditions and sex. Here, we show that MP rearing reduces basal proliferation of adult NSCs in both the SVZ and SGZ of prairie voles. Twenty-four hours of cohabitation with mating enhances NSCs proliferation in BP male voles; however, MP males exhibit fewer newborn cells in the SVZ and show no changes in SGZ NSCs in response to this socio-sexual stimulus. These differences parallel alterations in the global epigenetic profile of newborn cells, which may influence NSCs activation. Further studies are warranted to identify specific genes that are activated or repressed via epigenetic modifications in MP versus BP prairie voles. In particular, locus-resolved protein–DNA interaction assays (e.g., ChIP-qPCR) should be used to test promoter and/or enhancer occupancy at prioritized candidate loci.

In females, we did not detect significant differences in NSCs proliferative activity or in the enrichment of the evaluated histone marks. This pattern may reflect the temporal dynamics of the neuroendocrine response to male sensory cues and suggest that longer or hormonally primed exposure may be required to reveal robust changes in female neurogenic niches.

In the present study, we assessed the effect of paternal deprivation at an early proliferative stage of adult neurogenesis (24 h post-stimulation). Future work should incorporate additional timepoints, including 48 h and longer post-labeling intervals (e.g., ~15 and ~45 days), which correspond to critical phases of migration/survival and subsequent neuronal maturation and circuit integration, respectively [20,106].

Overall, our findings support the notion that monoparental rearing alters the adult neurogenic process and its epigenetic regulation in a sex- and niche-specific manner, with potential consequences for socio-sexual behavior and pair-bond formation.

## Figures and Tables

**Table 1:** Sexual behavior of biparentally (BP) and monoparentally (MP) reared male prairie voles.

Sexual Parameter	BP Males (n = 8)	MP Males (n = 10)
Mount latency (s)	568.9 ± 153.1	758.2 ± 254.7
Number of mounts	13.0 ± 3.3	13.9 ± 3.7
Intromission latency (s)	533.3 ± 145.8	766.9 ± 221.8
Number of intromissions	21.0 ± 2.9	19.2 ± 4.4
Ejaculation latency (s)	1697.2 ± 413.8	1419.8 ± 409.8
Number of ejaculations	1.3 ± 0.3	1.6 ± 0.4
Lordosis index *	0.94 ± 0.02	0.97 ± 0.013

Data are represented as mean ± SEM. No statistically significant differences were found for any analyzed parameter. Student *t*-test was used for variables that passed the normality test (number of mounts and intromissions, and lordosis index), while the Mann–Whitney U-test was applied to non-normally distributed data (mount, intromission, and ejaculation latency and number of ejaculations). * Stimuli females display high levels of lordosis (dorsiflexion of the spine); the ratio of dorsiflexions performed in response to the mounts received is shown (maximum score = 1).

**Table 2:** Partner preference test in BP and MP reared male and female prairie voles.

Parameter	BP Males (n = 8)	MP Males (n = 10)	BP Females (n = 8)	MP Females (n = 8)
Partner time	2701.5 ± 788.6	2730.1 ± 551.4	5930.2 ± 396.8 **	5357.0 ± 761.4 ^##^
Stranger time	4499.6 ± 788.6	4468.0 ± 551.3 ^+^	1073.7 ± 406.4 ***^, +++^	1852.5 ± 766.2 ^##, ++^
Preference score	0.375 ± 0.11	0.379 ± 0.07	0.848 ± 0.05 ***	0.744 ± 0.10 ^##^
% partner huddling	50%	40%	88%	25% °

Data are represented as mean ± SEM. Different from BP males: ** *p* < 0.01, *** *p* < 0.001; different from MP males: ^##^ *p* < 0.01. Different from BP females ° *p* < 0.05. Different from time spent with the partner: ^+^ *p* < 0.05, ^++^ *p* < 0.01, ^+++^ *p* < 0.001.

**Table 3:** Proliferative activity in the neurogenic niches of BP and MP reared male and female prairie voles.

Neurogenic Niche	Group	Cell Population	
		BrdU^+^	BrdU^+^/SOX2^+^
SVZ	BP Control	M < F (*p* = 0.009)	M < F (*p* = 0.02)
	BP Cohabitation	ns	M > F (*p* = 0.04)
	MP Control	M < F (*p* = 0.002)	M < F (*p* = 0.019)
	MP Cohabitation	ns	M > F (*p* = 0.03)
SGZ	BP Control	M > F (*p* = 0.01)	M > F (*p* = 0.004)
	BP Cohabitation	M > F (*p* < 0.001)	M > F (*p* < 0.001)
	MP Control	ns	ns
	MP Cohabitation	M > F (*p* = 0.03)	ns

A *t*-test was performed to compare the number of cells per mm^2^ between males (M) and females (F), ns = no statistical difference.

## Data Availability

The data presented in this study are openly available in [Zenodo] at https://doi.org/10.5281/zenodo.17781438, accessed on 1 December 2025.

## Abbreviations

The following abbreviations are used in this manuscript:

BDNF	Brain-Derived Neurotrophic Factor
BP	Biparentally reared subjects
BrdU	5′-bromo-2′-deoxyuridine
CA3	Cornu Ammonis region 3
DEGs	Differentially Expressed Genes
DG	Dentate Gyrus
GR	Glucocorticoid receptor
MP	Monoparentally reared subject
NSCs	Neural Stem Cells
OB	Olfactory Bulb
PI	Preference Index
PPT	Partner Preference Test
RAR	Retinoic Acid Receptor
RMS	Rostral Migratory Stream
SGZ	Subgranular Zone
SVZ	Subventricular Zone
TrkB	Tropomyosin receptor kinase B
